# Hemodynamic monitoring in the era of evidence-based medicine

**DOI:** 10.1186/s13054-016-1534-8

**Published:** 2016-12-20

**Authors:** Bernd Saugel, Manu L. N. G. Malbrain, Azriel Perel

**Affiliations:** 1 Department of Anesthesiology, Center of Anesthesiology and Intensive Care Medicine, University Medical Center Hamburg-Eppendorf, Martinistrasse 52, 20246 Hamburg, Germany; 2 Department of Intensive Care, Ziekenhuis Netwerk Antwerpen, Campus ZNA Stuivenberg, Antwerp, Belgium; 3 Department of Anesthesiology and Critical Care, Sheba Medical Center, Tel Aviv University, Tel Aviv, Israel

## Abstract

Hemodynamic instability frequently occurs in critically ill patients. Pathophysiological rationale suggests that hemodynamic monitoring (HM) may identify the presence and causes of hemodynamic instability and therefore may allow targeting therapeutic approaches. However, there is a discrepancy between this pathophysiological rationale to use HM and a paucity of formal evidence (as defined by the strict criteria of evidence-based medicine (EBM)) for its use. In this editorial, we discuss that this paucity of formal evidence that HM can improve patient outcome may be explained by both the shortcomings of the EBM methodology in the field of intensive care medicine and the shortcomings of HM itself.

Hemodynamic monitoring (HM) plays a central role in the care of critically ill patients, and yet the evidence that HM improves patient outcome is either small or, more often, non-existent [1, 2]. With the growing importance and influence of evidence-based medicine (EBM), it is imperative that we better understand the reasons for, and implications of, the absence of evidence for the way we use HM in the intensive care unit (ICU) and in the operating room.

Since its introduction in 1992, EBM has become a “holy grail” for rational clinical practice guided by scientific clinical research findings. EBM was meant to initiate a paradigm shift in clinical decision making which should be based on “formal rules of evidence evaluating the clinical literature” [3]. EBM also defined a hierarchy of evidence, whereby randomized controlled trials (RCTs) and systematic reviews and meta-analyses of RCTs are regarded as superior to animal, in vitro or observational studies, case reports, and expert opinions [3, 4]. Advocates of EBM emphasize that it combines individual clinical experience with the best available external evidence from systematic research facilitating decision making in the care of individual patients [4]. At the same time, EBM, by its own definition, de-emphasizes the importance of pathophysiologic rationale in making clinical decisions [3]. Such de-emphasis is of special relevance to HM monitoring in critical care and in anesthesia.

The hemodynamic status of critically ill patients is complex; it may include varying degrees of hypovolemia, left and right ventricular dysfunction, abnormalities of vascular tone, and microvascular dysfunction. These acute cardiovascular impairments are often further complicated by chronic co-morbidities. Physical examination and conventional HM cannot adequately assess the nature and extent of such hemodynamic dysfunction [5–7]. HM can therefore be viewed as a means to minimize the uncertainty that often surrounds the patient’s hemodynamic status. This physiological reasoning stands behind the recommendations for the use of advanced HM in high-risk surgical patients [8] and in critically ill patients in circulatory shock [9–11]. The discrepancy between these recommendations and the weakness of the associated evidence indicates that, in the absence of formal evidence as defined by the strict criteria of EBM, the basis of clinical practice relies on our best understanding of the underlying pathophysiological processes that are associated with critical illness.

The lack of evidence behind our practice of HM may be explained by the significant limitations of EBM. There is a shortage of relevant, high-quality evidence that shows a beneficial effect of any intervention on mortality in ICU patients [12]. One of the reasons for the frequent negative results of RCTs is the inclusion of heterogeneous patient populations with a wide range of disease syndromes [2, 13]. Many RCTs are designed with a “one-size-fits-all” approach, making the interventions potentially useful for some patients but harmful for others (“misalignment”). EBM has therefore been criticized for applying population-derived data to individual patients whose unique situation may be ignored. In the context of HM, some of the “negative” studies on the pulmonary artery catheter (PAC) may have included patients in whom the information provided by the PAC has been useful, while being misleading in others. Another pitfall of the “one-size-fits-all” approach in HM is the determination of fixed “goals” of resuscitation for a whole patient population, while disregarding the limitations and confounding factors of the physiological variables (and their specific values) that have been defined as goals [14]. These shortcomings of EBM in intensive care may explain the “decline of truth” effect; namely, that the original findings of “seminal” RCTs cannot be replicated or are refuted by later studies [15]. These significant inherent limitations of RCTs in ICU patients may make them inferior to well-designed observational studies for a variety of research questions [13].

However, the lack of evidence for our practice of HM may also be due to the inherent limitations of HM itself. First and foremost, the monitoring of physiological variables per se cannot improve outcome unless it is followed by correct therapeutic interventions. Making the “correct” decision is not always straightforward even when advanced HM is applied, due to the frequent misinterpretation of the monitored parameters, the failure to identify their relative importance, and the remaining uncertainty regarding the exact nature of the physiological impairment. The resulting variability of care precludes the assessment of the value of that particular monitoring modality per se by a formal RCT. In addition, each hemodynamic variable has inherent limitations and confounding factors. The monitoring of cardiac output (CO), for instance, may be crucial in many instances of hemodynamic instability. However, the CO value in and by itself may not necessarily lead to the “correct” therapeutic decision, since the optimal CO cannot always be determined: a high CO may not be high enough, and a low CO does not tell us what to do (e.g., fluids, inotropes?). The correct application of hemodynamic data necessitates the integration of various variables that may complement each other in order to provide the whole clinical picture. On the other hand, such a multiparametric approach to decision making based on HM may introduce another source of variability which can further complicate any study design that is aimed at establishing the value of HM in general, and of any specific variable in particular. In addition, HM may increase the tendency to normalize (or even maximize) the measured physiological variables, although “normalcy” of hemodynamic variables does not necessarily mean “adequacy”. Last but not least, monitoring technologies need to be meticulously validated with regard to their measurement performance before they should be used in clinical studies aiming to demonstrate an impact on outcome.

HM supplies invaluable insights about the patient’s hemodynamic status and is essential for the correct individual management of critically ill patients. The paucity of formal evidence showing that HM is improving patient outcome may be explained by both the shortcomings of EBM and HM itself. The honest and open acknowledgement of these shortcomings should become an integral part of the education of clinicians who take care of critically ill patients.

## Abbreviations

CO: Cardiac output
EBM: Evidence-based medicine
HM: Hemodynamic monitoring
ICU: Intensive care unit
PAC: Pulmonary artery catheter
RCT: Randomized controlled trial
